# Notes and News

**Published:** 1904-06-04

**Authors:** 


### NOTES AND NEWS.

LAST Saturday the Duchess of Albany laid the memorial stone of a Cottage Hospital for Walton, Hersham, and Oatlands. It is intended as a Coronation memorial and thanksgiving offering for the King's restoration to health. The cost is estimated at £3,200. Mr. E. Pettit will defray the cost of furnishing.

THE next International Congress of Physiologists will be held at Brussels in August.

MR. BRUCE CLARKE is a candidate for a seat on the Council of the Royal College of Surgeons of England.

THE Duke of Norfolk will present an ophthalmic department to the Norfolk and Norwich Hospital costing £1,000.

A NEW operating theatre has been opened at the Princess Alice Memorial Hospital, Eastbourne, presented by Mr. C. H. Evill.

A NEW infectious diseases hospital has been erected near Castle Douglas to serve several local authorities. Twelve beds have so far been provided in blocks for scarlet fever and typhoid patients. The cost is about £3,500.

THE last annual report of the Commissioners in Lunacy for Scotland comments on the increase in lunacy and the want of accommodation for the poorer class of private patient.

THERE is a good deal of activity in hospital construction in the United States. A new City and County Hospital has been opened at Huntsville, Alabama; the City of Hot Springs and the Ladies' Hospital Association of Pine Buffs are projecting hospitals. The new Providence Hospital at Oakland, California, has been opened. In Colorado the Denver St. Joseph's Hospital has been added to, and a Sanatorium at Glenwood Hills is proposed. In Connecticut the Bridgeport Hospital is to be enlarged.

AN endeavour will be made to establish a self-supporting convalescent home for Manchester.

DR. RICHARD CATON, of Liverpool, will deliver the Harveian Oration on June 21st, and on the same day the Harveian Festival of the Royal College of Physicians will take place.

THE Commemoration Day proceedings at Livingstone College, Leyton, will be of special interest this year owing to the opening of a small laboratory which has been arranged in the college, and special demonstrations will be given. Professor Sims Woodhead will preside at the preliminary meeting at 3.30 and afterwards open the laboratory.

THE health of the Russian Army in Manchuria is stated to be good. During the last week in May, only twenty-seven cases of infectious diseases were reported, and amongst them no cases of plague. The Czaritsa's hospital train left for the seat of war on May 26th. It consists of fourteen ambulance cars and two operating cars, and is supplied with bathrooms, *x*-ray compartment, dispensary, sterilising, distilling, and ice-making plant. A chapel and library are also provided. The staff consists of three medical men, four sisters of mercy, and three assistants. The Czaritsa furnished the whole train, and will provide £100 monthly to procure delicacies for the patients.

A CORRESPONDENT to the *Times* points out a very real case for reform in reference to the position now held by medical officers of health. The appointments are made yearly, the Provincial Boards and Councils are able to dismiss the medical officer independently of the Local Government Board, to whom it is his duty to report. A temporary appointment held at the pleasure of authorities who may not desire to adopt reforms, is not satisfactory as far as the protection of the public is concerned. There can be no continuity of experience nor independence of opinion under these circumstances, both of which undoubtedly are necessary for an efficient officer, and the sooner the Local Government Board remedies the existing arrangements the better. It is held that they have the power, if they choose to exercise it.

IN the House of Commons Mr. R. Cameron asked whether steps could be taken to prohibit the manufacture of serum in laboratories licensed under the Vivisection Acts, since it was admitted in the reports of the Lister Institute of Preventive Medicine that ill-effects sometimes resulted from injections of horse serum. Mr. Akers Douglas replied that the manufacture of serum was outside the Cruelty to Animals Act, and that he was not therefore prepared to act on the suggestion. He pointed out that the suggestion was apparently based upon remarks in a pamphlet published by the Lister Institute, which was intended to reassure the public if a rash or other symptoms followed inoculation by explaining that these symptoms were practically harmless and temporary. Mr. Akers Douglas remarked that he did not interpret the meaning of the pamphlet in the same way as Mr. Cameron.

